# O Desafio de Incorporar Tecnologias de Alto Custo: Uma Análise dos Inibidores de PCSK9

**DOI:** 10.36660/abc.20210840

**Published:** 2021-11-01

**Affiliations:** 1 Universidade Federal do Rio Grande do Sul Instituto de Avaliação de Tecnologia em Saúde RS Brasil Instituto de Avaliação de Tecnologia em Saúde - INCT/IATS, Universidade Federal do Rio Grande do Sul, RS - Brasil; 2 Universidade Federal do Rio Grande do Sul Programa de Pós-Graduação em Epidemiologia Porto Alegre RS Brasil Programa de Pós-Graduação em Epidemiologia - Universidade Federal do Rio Grande do Sul (UFRGS), Porto Alegre, RS - Brasil; 3 Universidade Federal do Rio Grande do Sul Programa de Pós-Graduação em Cardiologia Porto Alegre RS Brasil Programa de Pós-Graduação em Cardiologia - Universidade Federal do Rio Grande do Sul (UFRGS), Porto Alegre, RS - Brasil; 4 Hospital Nossa Senhora da Conceição Programa de Residência Médica em Medicina Interna Porto Alegre RS Brasil Programa de Residência Médica em Medicina Interna - Hospital Nossa Senhora da Conceição, Porto Alegre, RS - Brasil; 5 Universidade Federal de Pelotas Hospital Escola Pelotas RS Brasil Hospital Escola, Universidade Federal de Pelotas, Pelotas, RS - Brasil; 6 Universidade Federal do Rio Grande do Sul Hospital de Clínicas de Porto Alegre Porto Alegre RS Brasil Hospital de Clínicas de Porto Alegre, Universidade Federal do Rio Grande do Sul, Porto Alegre, RS - Brasil; 7 Hospital Moinhos de Vento Serviço de Cardiologia Porto Alegre RS Brasil Serviço de Cardiologia, Hospital Moinhos de Vento, Porto Alegre, RS - Brasil

**Keywords:** Gastos em Saúde, Análise de Custo-Benefício, Pró-Proteína Convertase 9/uso terapêutico, Avaliação de Tecnologia Biomédica, Aterosclerose, Sistema Único de Saúde (SUS)

Desde a descoberta em 2003 que mutações com perda de função no gene que codifica a pró-proteína convertase subtilisin-kexin tipo 9 (PCSK9) reduziam os níveis de colesterol LDL, há um interesse crescente no uso de vias da PCSK9 para tratar pacientes com risco cardiovascular aumentado e aterosclerose.^1,2^ Em múltiplos ensaios clínicos randomizados, os inibidores de PCSK9 (iPCSK9) reduziram os níveis de LDL, com redução significativa de eventos cardiovasculares, embora o efeito na mortalidade tenha sido menos consistente.^3^ O ensaio clínico FOURIER foi o maior dos ensaios clínicos randomizados com esses fármacos, tendo incluído 27.564 pacientes de alto risco e demonstrou redução de eventos cardiovasculares maiores com uso de iPCSK9, sem impacto significativo na mortalidade cardiovascular.^4^

Contudo, o alto custo da terapia, de uso contínuo por toda a vida, é um obstáculo importante à sua utilização. O custo impacta diretamente a prescrição desses fármacos pelos médicos, a adesão dos pacientes e a adoção em larga escala pelos sistemas de saúde.^5^ Este não é um problema exclusivo de países de baixa e média renda. Vários estudos internacionais apontaram que, na perspectiva econômica, os preços desses fármacos estavam desproporcionais ao benefício esperado.^6,7^ Houve grande apelo da comunidade internacional para que o preço dos fármacos fosse reduzido, o que vem acontecendo no decorrer dos anos.^7,8^

No Brasil, o cenário também é bastante crítico, pois essa classe não foi aprovada para incorporação no SUS, nem está prevista no rol da saúde suplementar. Nesse sentido, a análise de custo-efetividade do evolocumabe em pacientes com alto risco cardiovascular no contexto do SUS – Brasil^9^ é muito oportuna.

Os autores utilizaram uma coorte de pacientes atendidos em hospital público da Bahia em combinação com dados do estudo FOURIER, extrapolado para o período em 10 anos, em um modelo de redução de risco cardiovascular para projetar os eventos em uma coorte brasileira. A população é aquela de maior probabilidade de benefício do uso de iPCSK9 no contexto da dislipidemia não-familiar,^10^ pois inclui pacientes com síndrome coronariana aguda no último ano (57% com infarto agudo do miocárdio) e níveis de LDL > 100 mg/dL apesar do uso de atorvastatina e ezetimibe. Os autores demonstraram um custo adicional de R$189.619 e uma razão incremental de custo-efetividade superior a R$1 milhão por desfecho cardiovascular evitado.

Alguns aspectos metodológicos do estudo merecem ser apontados antes de interpretarmos os dados. A redução de eventos cardiovasculares foi uma extrapolação da redução prevista nos níveis de colesterol, na ordem de 35% de redução relativa e 12% na redução absoluta em 10 anos. Contudo, no estudo FOURIER, apesar de uma redução em 59% no colesterol LDL ocorreu uma redução relativa do desfecho primário de 15% e redução absoluta de apenas 1,5%.^4^ Certamente uma superestimação do benefício no modelo proposto pelos autores.

Quanto aos custos aplicados, foram utilizados custos diretos de aquisição dos medicamentos e reembolsos tabelados pelo SUS para internações por infarto agudo do miocárdio, acidente vascular cerebral e revascularização do miocárdio, ponderando pela frequência dos eventos observada no FOURIER. No entanto, os valores de reembolso praticados pelo SUS para estes procedimentos não são atualizados, representando valores frequentemente subestimados em comparação com os custos reais das internações.^11^

Adicionalmente, o custo da atorvastatina, baseado no valor da aquisição por hospital público de referência local, provavelmente é inferior ao custo da aquisição direta pelos pacientes, cenário plausível para análise sob a perspectiva da sociedade. O custo unitário do tratamento com evolocumabe não foi descrito, mas é conhecido que houve redução nos últimos anos do preço ao consumidor. Os autores optaram por apresentar os resultados na forma de custo por evento cardiovascular evitado; embora a decisão tenha mérito, o uso do desfecho medido em custo por ano ajustado para qualidade de vida é considerado o padrão-ouro, e permitiria comparação com outras terapias em saúde.^12^

Essas questões metodológicas demonstram o quanto são complexos e sensíveis os estudos desta natureza. Precisamos unir esforços para produção do conhecimento em análise econômica no cenário de saúde do Brasil, e nesse sentido congratulamos os autores.

Com esse tema cabe a reflexão de como podemos oferecer aos nossos pacientes terapias com valor clínico agregado, mas muito custosas. Os recursos são finitos e devemos priorizar terapias com boa relação de custo-efetividade. Ou seja, aquelas que trazem um maior benefício a um custo razoável. Para resolver esta equação, o caminho é maximizar a escolha de pacientes de mais alto risco e buscar redução de preços para os pacientes.^13^

Sabemos que as inovações tecnológicas estão na fronteira da nossa prática e queremos oferecer o máximo a quem precisa. Para isso, precisamos repensar nosso sistema e modelo médico-assistencial, reduzindo ineficiências e cortando desperdícios. Incorporar tecnologias de alto custo, principalmente para o controle das doenças cardiovasculares, depende de otimização do recurso existente, supressão de ações que não agregam valor ao paciente e pactuação de preços de acordo com o benefício esperado.^13^

O estudo^9^ se debruça sobre a custo-efetividade dos iPCSK9, encontrando razão incremental de custo-efetividade desfavorável para incorporação, com base nos parâmetros utilizados. O estudo representa um passo na direção da ampliação do papel das análises econômicas para a tomada de decisão no sistema de saúde brasileiro. Para oferecer as melhores intervenções aos nossos pacientes, devemos almejar por mais estudos econômicos, melhorando a compreensão do papel dos iPCSK9 e de outras terapias de alto custo.
