# Supplementary material for: O Desafio de Incorporar Tecnologias de Alto Custo: Uma Análise dos Inibidores de PCSK9
Source: Arq Bras Cardiol. 2021 Nov 1;117(5):997–8. [Article in Portuguese] doi: 10.36660/abc.20210840 (PMC8682091; doi:10.36660/abc.20210840)
Supplement: Translation

# Translation: The Challenge of Incorporating High-Cost Technologies: An Analysis of PCSK9 Inhibitors

**Affiliations:** 1 RS Brasil Instituto de Avaliação de Tecnologia em Saúde - INCT/IATS, Universidade Federal do Rio Grande do Sul, RS - Brasil; 2 Porto Alegre RS Brazil Programa de Pós-Graduação em Epidemiologia - Universidade Federal do Rio Grande do Sul (UFRGS), Porto Alegre, RS - Brazil; 3 Porto Alegre RS Brazil Programa de Pós-Graduação em Cardiologia - Universidade Federal do Rio Grande do Sul (UFRGS), Porto Alegre, RS - Brazil; 4 Porto Alegre RS Brazil Programa de Residência Médica em Medicina Interna - Hospital Nossa Senhora da Conceição, Porto Alegre, RS - Brazil; 5 Pelotas RS Brazil Hospital Escola, Universidade Federal de Pelotas, Pelotas, RS - Brazil; 6 Porto Alegre RS Brazil Hospital de Clínicas de Porto Alegre, Universidade Federal do Rio Grande do Sul, Porto Alegre, RS - Brazil; 7 Porto Alegre RS Brazil Serviço de Cardiologia, Hospital Moinhos de Vento, Porto Alegre, RS - Brazil

**Keywords:** Health Expenditures, Cost-Bebefit Analysis, Proprotein Convertase/therapeutic use, Technology Assessment, Biomedical, Atherosclerosis, Unified Health System

Since the discovery, in 2003, that loss-of-function mutations in the gene that encodes proprotein convertase subtilisin/kexin type 9 (PCSK9) reduce LDL cholesterol levels, there has been growing interest in using PCSK9 pathways to treat patients with increased risk of cardiovascular disease and atherosclerosis.^1,2^ In multiple randomized clinical trials, PCSK9 inhibitors reduced LDL levels, with a significant reduction in cardiovascular events, although the effect on mortality has been less consistent.^3^ The FOURIER clinical trial was the largest randomized clinical trial with these medications, including 27,564 patients with high risks; it demonstrated a reduction in major cardiovascular events with the use of PCSK9 inhibitors, without any significant impact on cardiovascular mortality.^4^

Nevertheless, the high cost of therapy, with continuous lifelong treatment, is an important obstacle to its use. Costs directly impact the prescription of these medications by physicians, as well as patient compliance, and large-scale adoption by health systems.^5^ This problem is not unique to low- and middle-income countries. Several international studies have indicated that, from an economic perspective, the prices of these drugs were not proportionate to the expected benefit.^6,7^ There has been massive appeal from the international community for a reduction in the price of PCSK9 inhibitors, which has been taking place over the years.^7,8^

In Brazil, this scenario is also rather critical, given that this class of drugs has not been approved for incorporation into the Brazilian Unified Health System (SUS), nor is it included in private health insurance coverage. Accordingly, the cost-effectiveness analysis of evolocumab in patients with high cardiovascular risks in the context of SUS in Brazil^9^ is very timely.

The authors used data from a cohort of patients treated at a public hospital in the Brazilian state of Bahia in combination with data from the FOURIER study, extrapolated for a period of 10 years, in a cardiovascular risk reduction model that simulates events in a Brazilian cohort. The population is the one most likely to benefit from the use of PCSK9 inhibitors in the context of non-familial dyslipidemia,^10^ consisting of patients treated for acute coronary syndrome in the last year (57% with acute myocardial infarction) and LDL levels > 100 mg/dL, notwithstanding the use of atorvastatin and ezetimibe. The authors demonstrated an additional cost of 189,619 Brazilian reais (BRL) and an incremental cost-effectiveness ratio greater than 1 million BRL per cardiovascular outcome avoided.

Some methodological aspects of the study should be pointed out before interpreting the data. The reduction in cardiovascular events was extrapolated from the predicted reduction in cholesterol levels, resulting in nearly 35% relative reduction and 12% absolute reduction over 10 years. However, in the FOURIER study, in spite of a 59% reduction in LDL cholesterol, there was a 15% relative reduction in the primary outcome and an absolute reduction of only 1.5%.^4^ Surely, the proposed model overestimates the benefit of therapy.

Regarding the applied costs, the authors used direct costs of acquiring the medications, and reimbursements tabulated by SUS for hospital admissions due to acute myocardial infarction, stroke, and myocardial revascularization, considering the frequency of events observed in FOURIER. However, the reimbursement values used by SUS for these procedures have not been updated, thus representing values that are frequently underestimated when compared to the actual costs of hospital admissions.^11^

Additionally, the cost of atorvastatin, based on the cost of acquisition by a local public reference hospital, is probably lower than the cost of direct acquisition by patients, which is a plausible scenario for an analysis from the societal perspective. The unit cost of treatment with evolocumab has not been described, but it is known that the consumer price has reduced over the past years. The authors opted to present the results in the form of cost per cardiovascular event avoided; although this decision holds some merit, measuirng the outcomes in cost per quality-adjusted life year is considered the gold standard, and it would allow comparison with other health therapies.^12^

These methodological issues demonstrate how complex and sensitive studies of this nature are. We need to join efforts to produce knowledge related to economic analysis in the Brazilian health scenario, and in this sense we congratulate the authors.

Regarding this topic, it is necessary to reflect on how we may offer our patients therapies with added clinical value, which are, however, very costly. Resources are finite, and we must prioritize cost-effective therapies, that is, those that offer the greatest benefit at a reasonable cost. To solve this equation, the path involves maximizing choices of patients with the highest risks and seeking to reduce prices for patients.^13^

We know that technological innovations are at the frontier of our practice, and we want to offer the best to those who need it. In order to do so, we need to rethink our healthcare system and model, reducing inefficiencies and cutting down on misspending. Incorporating high-cost technologies, especially to control cardiovascular diseases, depends on optimizing existing resources, suppressing actions that do not add value for patients, and agreeing on prices according to the expected benefits.^13^

The study^9^ contemplates the cost-effectiveness of PCSK9 inhibitors, finding an unfavorable incremental cost-effectiveness ratio for incorporation, based on inputted parameters. The study represents a step toward expanding the role of economic analysis for decision-making in Brazilian health system. To offer the best interventions to our patients, we should aim for more economic studies, improving our understanding of the role of PCSK9 inhibitors and other high-cost therapies.
